# Regional Immunotherapy for Peritoneal Carcinomatosis in Gastroesophageal Cancer: Emerging Strategies to Re-Condition a Maladaptive Tumor Environment

**DOI:** 10.3390/cancers15205107

**Published:** 2023-10-23

**Authors:** Catherine R. Lewis, Neda Dadgar, Samuel A. Yellin, Vera S. Donnenberg, Albert D. Donnenberg, David L. Bartlett, Casey J. Allen, Patrick L. Wagner

**Affiliations:** 1 Allegheny Health Network Cancer Institute, Pittsburgh, PA 15212, USA; catherine.lewis@ahn.org (C.R.L.); donnenbergad@ahn.org (A.D.D.); david.bartlett@ahn.org (D.L.B.); casey.allen@ahn.org (C.J.A.); 2 Cole Eye Institute, Cleveland Clinic, Cleveland, OH 44195, USA; dadgarn@ccf.org; 3 Department of Surgery, Lehigh Valley Health Network, Allentown, PA 18101, USA; samuel.yellin@lvhn.org; 4 Department of Cardiothoracic Surgery, University of Pittsburgh School of Medicine, Pittsburgh, PA 15213, USA; donnenbergvs@upmc.edu; 5 Hillman Cancer Centers, University of Pittsburgh School of Medicine, Pittsburgh, PA 15213, USA

**Keywords:** gastric cancer, peritoneal metastasis, carcinomatosis, immunotherapy

## Abstract

**Simple Summary:**

Regional immunotherapy is a promising approach for treating peritoneal carcinomatosis gastric/gastroesophageal junction cancer (GC-PC) patients. This review explores the unique characteristics of GC-PC and peritoneal immune biology that make it a suitable target for immunotherapy. We discuss pre-clinical and clinical trials exploring a variety of potential immunomodulatory regional strategies designed to alter the peritoneal immune environment. Lastly, we present a blueprint for a future combinatorial strategy to leverage the peritoneal immune environment toward improved efficacy of regional cellular therapy, with the goal of creating durable local and systemic responses in GC-PC.

**Abstract:**

Peritoneal carcinomatosis originating from gastric/gastroesophageal junction cancer (GC-PC) occurs in a defined subset of gastric cancer patients with unique clinical, pathologic, molecular and immunologic characteristics that create significant obstacles to effective treatment with modern therapy. Although systemic chemo- and immuno- therapy have yielded disappointing results in GC-PC, recent advances in the characterization of GC-PC and peritoneal immune biology present new opportunities for targeted therapeutics. In this review article, we discuss the distinct properties of GC-PC and the peritoneal immune environment as they pertain to current and investigative treatment strategies. We discuss pre-clinical studies and clinical trials relevant to the modulation of the peritoneal environment as a therapeutic intervention in GC-PC. Finally, we present a road map for future combinatorial strategies based on the conception of the peritoneal cavity as a bioreactor. Within this isolated compartment, prevailing immunosuppressive conditions can be altered through regional interventions toward an adaptive phenotype that would support the effectiveness of regionally delivered cellular therapy products. It is hoped that novel combination strategies would promote efficacy not only in the sequestered peritoneal environment, but also via migration into the circulation of tumor-reactive lymphocytes to produce durable systemic disease control, thereby improving oncologic outcome and quality of life in patients with GC-PC.

## 1. Introduction

Peritoneal carcinomatosis (PC) arising from gastric/gastroesophageal junction cancer (GC) presents a complex and challenging clinical scenario, with devastating manifestations of intestinal inflammation, obstruction, and nutritional compromise. Outcomes are uniformly poor in afflicted patients, and there is an urgent need for innovative approaches to address the unique patient care challenges it poses [1,2]. Although PC can arise from any number of primary tumor types, immunotherapy is of special interest in gastric cancer peritoneal carcinomatosis (GC-PC), given the rapid integration of immune checkpoint inhibition into the care of patients with GC. In this review, we discuss GC-PC as a distinct clinicopathologic entity with distinct molecular and biologic characteristics and propose a combination therapy paradigm that integrates multiple interventions in treating this unique subset of GC. We explore the rationale for and challenges inherent in achieving a durable adaptive immune response to GC-PC. Finally, we discuss past clinical trials focused on immunotherapy in GC-PC and a template for future combinatorial regimens.

## 2. Current Treatment of Peritoneal Carcinomatosis in Gastric Cancer

Current therapeutic strategies for GC-PC have limited efficacy. GC-PC can arise synchronously or metachronously, and current guidelines recommend diagnostic laparoscopy during the staging process in order to detect and quantify the extent of peritoneal disease (peritoneal cancer index (PCI)) [3,4,5]. Systemic palliative chemotherapy is the standard of care and has yielded consistently disappointing oncologic outcomes, with a significant majority of patients experiencing progressive intestinal obstruction, ascites, malnutrition, and cachexia [6,7]. Primary systemic treatment regimens include platinum-based and fluoropyrimidine-containing compounds such as FOLFOX, XELOX, ECF, FLOT, and S-1/cisplatin [3,4], and systemic immunotherapy as the primary therapeutic approach for metastatic GC [8]. Despite the addition of anti-PD-1 immunotherapy to systemic therapy following the CheckMate 649 clinical trial, overall survival or disease-free survival benefits are modest [9], most likely due to the significant redundancy in immunoregulatory pathways.

Systemic immune checkpoint inhibition (ICI) has not been fully studied as an intervention specific to this subset of GC-PC. Insights about efficacy can be gained, however, by examining the results of ICI therapy in studies for advanced gastric cancer that included patients with GC-PC, as has been recently reviewed [10]. In the ATTRACTION-2 study, comparing nivolumab vs. placebo in patients with advanced GC [11], 17% of the 493 randomized patients had peritoneal metastatic disease (84 patients). The primary endpoint was overall survival (OS). Post-hoc analysis did not identify a benefit in the subgroup with peritoneal disease (HR 0.74, [95% CI 0.48 - 1.15]). However, it is important to note that given the limited number of patients with peritoneal disease, the post-hoc analysis carried only 10% power to reject the null hypothesis of no difference at an effect size seen in the overall group (HR 0.65). The OS rates of nivolumab vs. placebo were 27.3% and 11.6% at 12 months, and 10.6% and 3.2% at 24 months, respectively [12]. Further studies are underway to determine baseline characteristics associated with long-term survival with nivolumab [13].

In the ATTRACTION-4 study, 724 patients with advanced, unresectable, or recurrent gastric cancer were randomized to chemotherapy plus nivolumab vs. chemotherapy alone, and 46% of the patients had peritoneal metastases. Among all patients, progression-free survival was significantly improved in the chemotherapy/nivolumab arm vs. the chemotherapy/placebo arm (HR 0.68 (98.51% CI 0.51–0.90); *p* = 0.0007). Peritoneal disease, however, was an important predictor of *non*-response (HR 0.51 vs. 1.04, 0.37–0.69 vs. 0.76–1.44, *p* = 0.0013). There was no improvement in OS. Given the large number of patients (333) with GC-PC in this study, it provided convincing evidence that the progression benefit of nivolumab was restricted to those without PC; on the contrary, in the OS analysis, a trend toward worse outcomes with nivolumab versus placebo was seen in patients with GC-PC (HR, 1.20, 95% CI, 0.94–1.53) [14]. A limitation of this study is that it was only performed in patients with HER2-negative, unresectable GC.

Ascites, in particular, has been found to be associated with very poor results in ICI-treated GC patients, who might otherwise be predicted to have favorable response rates to ICI based on predictive biomarkers. In a retrospective analysis of 59 MSI-H/dMMR GC patients undergoing anti-PD-L1 and/or anti-CTLA-4 therapy at 12 international centers, patients with GC-PC achieved outcomes comparable to those with non-peritoneal metastases (aHR 1.87, 95% CI, 0.64–5.46; aHR 2.15, 95% CI, 0.64–7.27). Patients with ascites, however, showed distinctly unfavorable progression-free (aHR 3.83, 95% CI 1.68–8.72) and overall (aHR 3.44, 95% CI 1.39–8.53) survival outcomes [15]. Taken together, these data suggest that for patients with GC-PC and ascites, the use of systemic ICI therapy without taking additional steps to condition the peritoneal cavity may be futile in improving outcomes for GC-PC patients.

Given poor results with systemic therapy, and success with cytoreductive surgery (CRS) in other gastrointestinal cancers, there is ongoing interest in defining the potential for curative surgical resection in GC-PC cases. However, when considering surgical intervention in GC-PC patients, it is essential to select patients carefully: only those with favorable performance status, evidence of disease stabilization after extensive first-line systemic therapy, and less extensive peritoneal disease with a high probability of complete cytoreduction are likely to derive benefit from this intensive approach. Recent research indicates that combining CRS with hyperthermic intraperitoneal chemotherapy (HIPEC) might improve the prognosis for a subset of GC-PC patients. Frequently used agents include docetaxel, oxaliplatin, cisplatin, doxorubicin, and mitomycin C, although consensus on a standardized and validated strategy remains elusive [3,16]. The most influential prognostic factors are the extent of peritoneal disease, response to systemic therapy, patient performance status, and completeness of cytoreduction (CC) [3,16]. A standout phase III study by Yang et al. found that patients undergoing CRS with HIPEC had a median survival of 11.0 months, versus 6.5 months for those undergoing CRS alone [17]. Although these promising results may show evidence of a benefit in highly selected patients, the contribution of CRS and intraperitoneal chemotherapy will likely plateau until more effective systemic and/or regional chemotherapy and immunotherapy options emerge.

## 3. Peritoneal Carcinomatosis as a Distinct Subtype of Gastric Cancer

Poor efficacy outcomes of systemic therapy in GC-PC have led investigators to study the differences between this subset of patients versus those with non-metastatic disease or with hematogenous or nodal metastases. Interestingly, peritoneal and solid organ (e.g., lung/liver) metastases are often mutually exclusive in GC patients, reinforcing the concept of PC as a distinct biologic subtype [18,19]. Moreover, large North American institutional series [20] and population-based registry studies [21] in Asian [22] and European [18,23,24,25] countries have indeed confirmed the presence of unique tumor and patient characteristics associated with this cohort of patients. Demographic and clinical characteristics of GC-PC include younger age, female sex, primary tumor location within the distal (non-cardia) stomach, signet cell/diffuse type histologic subtype, advanced T and N stages, and lymphovascular invasion. Among GC patients, GC-PC is associated with poor prognosis [5,21], compromised nutritional [26], and global performance status [27].

The biological processes inherent in peritoneal metastases may account for the differences between the clinical and pathologic profiles of patients with GC-PC versus those of patients with non-metastatic GC or GC metastatic to other visceral sites [18]. These mechanisms include transmural invasion, lymphatic and hematogenous dissemination to the peritoneal cavity, adhesion to the mesothelial lining of the visceral and parietal peritoneum, invasion and outgrowth in the peritoneal metastatic niche, and evasion of the peritoneal immune system [28]. Molecular signatures consistent with epithelial-to-mesenchymal transition [29], enhanced cell adhesion capability [30], angiogenesis [31], tissue remodeling [32], and immunosuppression [33,34] are found in GC-PC and may predict peritoneal recurrence. For example, Takeno et al. (2010) described a 22-gene expression profile from primary GC tissue associated with peritoneal recurrence following curative resection for gastric cancer [35], while Lee et al. (2021) identified a similarly predictive 12-gene profile [36]. A prediction score for peritoneal recurrence based on the immune cell infiltrate of the primary gastric tumor was developed by Zhang et al. [37], who reported that mast cells, effector memory T cells, interstitial and plasmacytoid dendritic cells (DCs), γδ-T cells, NK cells, macrophages, CD8+ T cells, and eosinophils were predictive of peritoneal recurrence, whereas Th_2_, T_reg_, NK_CD56-dim_ cells, and activated DCs were predictive of a lack of peritoneal recurrence.

More recently, there has been intense interest in the comprehensive characterization of GC-PC as a distinct molecular subset of GC. In a pair of seminal works by Ajani and colleagues, distinctive molecular signatures from mutational, transcriptomic, and proteomic analyses have been assigned to GC-PC versus primary GC tissue. These signatures included high rates of CDH1 and TP53 mutations, 6q loss and chr19 gain, chromosomal instability, and transcriptional upregulation of genes associated with cell cycle and immune tolerance. The demonstration of a “mesenchymal-like” subset of GC-PC, expressing epithelial to mesenchymal transition (EMT) phenotype and morphology with high expression of the checkpoint protein TIM-3 and its ligand (galectin-9), as well as TGF-β, reinforces the concept of immune evasion and tumor EMT as central features of GC-PC [38,39].

Taken together, the clinical, pathologic, and molecular features of GC-PC not only define this as a unique, chemo-resistant subpopulation of GC patients but may also meaningfully influence the rational development of novel targeted or immuno-therapeutic strategies for this group. For example, Tanaka et al. profiled paired primary GC and GC-PC lesions in search of therapeutic targets, finding over-representation of receptor tyrosine kinase and MAP kinase pathway alterations in the metastatic lesions relative to their corresponding primary tumors; half of the genetic alterations detected in GC-PC were potentially targetable [40]. In an analysis of matched primary GC, GC-PC, and non-neoplastic gastric tissue samples, Lim et al. documented the enrichment within GC-PC of mutations in metastasis-associated genes (including L1CAM and TGFBR1), along with druggable genes such as BRAF, ERBB4, FLT3, PIK3CA, and PIK3C2B [41]. These findings present exciting opportunities for the strategic addition of targeted immuno-therapy to standard systemic or surgical options for GC-PC.

Lastly, patients with GC-PC will have unique clinical characteristics that may impact the tolerance of aggressive combination regimens. For example, secondary intestinal involvement and nutritional compromise could render GC-PC patients especially susceptible to digestive system toxicity during treatment. Likewise, while the disproportionately younger and female set of patients with GC-PC may have a favorable performance status and eligibility for immunotherapy, they may also be less responsive or more susceptible to immune-related adverse events (irAEs) due to biological sex itself as well as higher rates of underlying autoimmune disorders [42,43]. Clearly, much work remains to translate the benefits of this emerging molecular understanding of GC-PC into new therapeutic options.

## 4. Peritoneal Cavity as a Distinct Immune Environment with Treatment Implications

Just as the tumor molecular characteristics of GC-PC present a substantial opportunity for rational and targeted approaches, a nuanced understanding of the peritoneal environment within which GC-PC exists will be essential to devising effective immunotherapy. The immune environment of GC-PC consists of the visceral and parietal peritoneal surfaces, along with the soluble and cellular immune constituents of peritoneal fluid. Each of these components is likely a key contributor to the dynamic interaction between tumor cells, the peritoneal mesothelium, and the immune system, with significant implications for the treatment of GC-PC. Here, we review the anatomic and physiologic principles defining the peritoneal cavity as they pertain to immunotherapy for GC-PC.

The mesothelial lining is traditionally conceptualized as a protective lining with repair and regenerative capacity, whereas, in reality, it is a physiologically dynamic tissue with regional functional variations. For example, differential gene expression and functional profiles of the visceral and parietal peritoneum have been documented and extensively reviewed [44]. Mesothelial cells are critical for peritoneal lining maintenance and repair, including secreting and responding to cytokines, chemokines, and growth factors. Mesothelial cells also contribute to adhesion and repair following trauma, capitalizing on a ready ability to undergo EMT in response to TGF-α derived from GC-PC cells [45,46]. Mesothelial cells express toll-like receptors (TLRs) that, upon recognizing pathogen-related molecules, can rapidly mobilize an innate immune response via NF-κB-mediated chemokine transcription [47]. An emerging concept of the “cancer-associated mesothelial cell” has evolved from recent studies in ovarian cancer in which intricate positive feedback circuits between cancer cells and adjacent mesothelial cells, mediated by TGFβ, directly contribute to tumor colonization, invasion, and immune evasion [47]. A senescent mesothelial cell phenotype, again mediated by TGF-β, may be central in promoting tumor growth and survival in GC-PC [30,48]. Other mesenchymal cell types found in the peritoneal lining also upregulate cytokine production when in close contact with GC-PC cells, as has been demonstrated for IL-6 from omental adipocytes [49] and IL-17 from mast cells [50]. A recurring theme in this area is the critical role of TGF-β as a signaling molecule important in physiologic repair and regeneration processes in the peritoneal cavity. In the context of GC-PC, however, TGF-β instead initiates maladaptive immunosuppressive and tumor-promoting processes, providing an unexplored opportunity for therapeutics targeted at this critical pathway [51,52].

The normal, uninflamed peritoneal cavity contains a cytokine and immune cellular profile that is quite distinct from the systemic circulation [53]. Kubicka et al., in a study of patients undergoing elective surgery for benign non-inflammatory gallbladder disease, found that 45% of peritoneal immune cells were CD68+ (monocytes/macrophages), 45% lymphocytes, 8% natural killer (NK) cells, and 2% B cells. The CD4:CD8 ratio in peritoneal T cells was 0.4, the inverse of what is well documented in the peripheral blood, with CD45RO-positive CD4 and CD8 memory cells being the prevalent phenotype [54]. More recent studies in malignant ascites and effusions from GC patients have confirmed that T cells are skewed toward a memory phenotype (CD45RO+ PD-1dim). However, T_reg_-associated phenotypes (CD69, CD25, and Foxp3) were also found, with each distinct subset carrying an independent prognostic value [55,56,57]. Further, tumor-promoting macrophage subsets have also been documented, both in peritoneal washings and in malignant ascites [58]. Eum et al., reported that tumor-associated macrophages (TAMs) derived from GC-PC ascites interact directly with the carcinoma cells, further driving the inflammatory milieu that favors tumor growth and invasion and is responsible for poor prognosis. TAM-derived IL-6, epidermal growth factor (EGF), and vascular endothelial growth factor (VEGF) have been identified as potential drivers of tumor progression in GC-PC patients [59,60].

The soluble protein milieu in the peritoneal cavity, commonly referred to as the peritoneal secretome, has been extensively characterized in PC from gastric and other malignancies. Vlaeminck-Guillem et al., reported significantly elevated levels of IL-6, IL-8, IL-10, TNF-α, and sICAM in the peritoneal secretome relative to serum from patients with PC and relative to peritoneal samples from control (non-cancer diagnosis) individuals. Additionally, cytokine levels were also found to correlate with peritoneal disease volume [61]. Wagner et al., comparing pan-cytokine peritoneal levels in patients undergoing surgery for malignancy with those undergoing surgery for benign non-inflammatory indications, documented key differences between the peritoneal secretome and serum with exorbitant concentrations of the cytokine IL-6 and its soluble receptor (sIL-6Rα) in malignant peritoneal fluid [62]. IL-6, when bound to sIL-6Rα, generates a ligand-receptor complex that can subsequently bind to cell-associated Gp-130 (IL-6Rβ), initiating trans-signaling in any cell that expresses Gp-130. This trans-signaling through the IL-6/IL-6R pathway could be a key driver of the maladaptive immune response in PC and a potential therapeutic target [63,64,65]. Other studies specific to GC-PC have consistently identified elevated IL-6 levels as a potential negative predictor [66], along with VEGF-α and IL-10 [55].

The overall picture of the peritoneal immune environment is that of a unique anatomical compartment poised for an immune reaction against microbial invasion from the gut, regeneration in the context of normal maintenance of the mesothelium, and repair of organ injury [64]. Because cavitary immune and repair/regenerative pathways drive tumor progression and metastasis [64,65], the peritoneal cavity may be especially conducive to harboring invasive carcinoma cells from gastric and other intra-abdominal tumors and supporting outgrowth and invasion into peritoneal tissues. Unlike other organs or cavities, the peritoneal potential space does not have a discrete draining lymph node basin, isolating it from mechanisms known to generate adaptive immunity. In this local environment, macrophages are polarized toward an M2 phenotype, where they participate in peritoneal tumor dissemination and growth [67]. Similarly, T-cells recruited into this environment are driven toward a pro-inflammatory Th2 phenotype, as they are in poor-prognosis primary gastric cancers [68]. In peritoneal malignancies, this creates an overall advantage for the tumor, which can condition the peritoneal environment and recruit T-cells, macrophages, and mesothelial cells to create a stable, self-perpetuating pro-inflammatory milieu that promotes EMT and local immunosuppression [64]. The implications for the treatment of GC-PC are clear: *regional* therapeutic interventions may prove successful where *systemic* immune interventions have failed to impact the sequestered and immunologically distinct peritoneal environment.

The unique properties of the peritoneal cavity provide a number of theoretical advantages for regional immunotherapy [69,70,71,72,73]. The mesothelium extends as a monolayer over the entire peritoneal surface [74], forming tight junctions that sequester locally secreted proteins in a bio-reactor-like fashion. We theorize that the contained nature of the peritoneal cavity can be leveraged to a therapeutic advantage if the peritoneal environment is appropriately conditioned with anti-cytokine antibodies and/or exogenous cytokines [75]. Moreover, while it may be difficult or impossible to re-condition the peritoneal environment using systemic therapy alone, the opposite may not be true. For example, intra-cavitary therapeutic strategies to alter the cellular and soluble immune constituents within the peritoneal cavity may have the potential to stimulate local anti-tumor responses that could propagate systemically and improve long-term outcomes. The peritoneal cavity, and specifically malignant ascites, is a rich source of cytotoxic lymphocytes that could also be harvested, expanded ex vivo, and re-administered to patients along with other complimentary interventions to alter the suppressive polarity of the environment [65]. Since these lymphocytes can traffic out of the peritoneal cavity into the systemic circulation, we envision that cavitary interventions to stimulate and support anti-tumor responses might also allow tumor-reactive T cells to migrate to extra-peritoneal sites of disease [64,65].

Given the unique and complex environment of the peritoneal cavity, it is unlikely that single-agent regional therapy will be able to provide a significant benefit over existing systemic therapeutic strategies. Designing a multi-modal approach incorporating peritoneal immune repolarizing strategies, adoptive cellular transfer, and tumor-targeted therapeutics combined with systemic immuno-oncology regimens will likely be critical to an effective and durable anti-tumor therapy [64]. Delivering immunotherapeutic interventions to the peritoneal cavity could also avoid systemic toxicity [65]. Although irAEs are a major source of morbidity in patients with advanced cancer, and direct localized toxic effects are certainly possible within the peritoneal cavity (peritonitis, abdominal infection, delayed wound healing), none of these have been reported to date [76].

## 5. Clinical Trials Reporting Immunotherapy Approaches for Gastric Cancer Peritoneal Carcinomatosis

In this section, we provide an overview of completed clinical studies examining the use of immunotherapy for patients with GC-PC, along with a discussion of ongoing active clinical trials in this area (Table 1).

### 5.1. Systemic Checkpoint Inhibition

Several case reports have described the use of systemic immunotherapy specifically for the treatment of GC-PC. Takami et al. presented a case of a patient with metachronous GC-PC four years following curative resection and adjuvant therapy with S-1 and cisplatin stage IV gastric cancer, at which time nivolumab was used and achieved a complete clinical response for an additional 3 years [83]. Similarly, Kuhara et al., also presented a response to nivolumab for GC-PC, along with improved quality of life and performance status in a patient treated for 20 months [84]. Nivolumab has also proven successful in downstaging gastric cancer. Toyota et al. presented a case of a patient with GC-PC in which two initial lines of systemic therapy were ineffective. The patient was then switched to nivolumab for 12 cycles. At subsequent laparotomy, a complete pathologic response was noted in the peritoneal disease, although the primary tumor responded poorly [85]. The potential for durable antitumor effects of nivolumab after discontinuation was demonstrated by Doi et al., who reported using nivolumab as third-line treatment in a patient with GC-PC, resulting in a complete radiographic response after 15 cycles and durability for an additional 10 months following discontinuation of therapy due to immune-related adverse events [86]. Likewise, Komo et al. also demonstrated resolution of peritoneal dissemination after nine courses of treatment and durability of response for an additional five months following treatment discontinuation for irAEs [87].

Combining systemic checkpoint inhibition with conventional systemic and intra-peritoneal chemotherapy is also being explored as a potential treatment regimen for GC-PC. NCT05648487 is a phase II study at Guangzhou Medical University evaluating the addition of a PD-1/PD-L1 antibody (sintilimab) in combination with systemic chemotherapy (SOX) and multi-cycle HIPEC with mitomycin, docetaxel, and oxaliplatin therapy in patients with histologically confirmed HER2-negative GC-PC with a PCI < 20. The study, with an intended enrollment of 46 patients, plans to evaluate the rate of R0 resection, with secondary outcomes of overall, event-free, and relapse-free survival as well as an objective response rate. The DRAGON-09 study, a phase II study in Shanghai at Ruijin Hospital (NCT05204173), is recruiting GC-PC patients to be treated with neoadjuvant sintilimab, paclitaxel, and S-1. The intended enrollment is 30 patients with confirmed PC and no other sites of distant metastases, with a primary endpoint of one-year survival. Secondary endpoints are adverse events, R0 resection rate, three-year OS, and three-year progression-free survival. If successful, these investigational regimens could improve outcomes in GC-PC, by improving quality of life related to the digestive morbidity of GC-PC and rendering patients more likely to be eligible for and benefit from CRS [77].

### 5.2. Intraperitoneal Immunotherapy

Completed studies of intraperitoneal immunotherapy in GC-PC are scarce. Catumaxomab is a bispecific/trifunctional antibody drug with binding sites to EpCAM, CD3 lymphocytes, and Fcγ receptors on antigen-presenting and other innate immune cells. The intended mechanism of action is to tether CD3-positive T cells to malignant epithelial cells in the peritoneal cavity to facilitate specific anti-tumor cytotoxicity. Initial studies were solely focused on safety and were performed without immune checkpoint blockade, limiting any conclusions about efficacy [88,89]. Encouraging results in populations with malignant ascites from a mixed group of underlying primary tumor histology led to a follow-up study to determine a potential oncologic benefit in GC patients [90,91]. A phase II study of perioperative IP catumaxomab infusion in patients undergoing surgery for GC demonstrated feasibility and safety in the adjuvant setting without an efficacy endpoint [92]. In a small study of GC-PC patients (*n* = 31), intraperitoneal therapy with catumaxomab and systemic chemotherapy, versus chemotherapy alone, did not produce a difference in progression-free or overall survival [76]. Two additional European studies, CatuNeo (NCT01504256) and IIPOP (NCT01784900), were planned to examine the use of catumaxomab in GC-PC, although both closed prematurely because of study drug unavailability. Currently withdrawn from U.S. and European regulatory and marketing pipelines, this agent is now undergoing a multinational Asian phase III study in GC-PC (NCT 04222114), with promising findings reported in the study’s initial pharmacokinetic and safety cohort [78]. This two-stage, multi-center, open-label, randomized controlled trial will evaluate the pharmacokinetics and safety of catumaxomab as an intra-peritoneal infusion (first stage) and subsequently evaluate the overall survival in patients randomized to catumaxomab infusion or investigator-choice intraperitoneal infusion (second stage). The study aims to evaluate the progression-free survival, objective response rate, clinical benefit rate, and adverse reactions in approximately 300 GC-PC patients who have failed at least two prior standard treatment regimens [78].

Intra-peritoneal cytokine blockade may provide durable modulation of the regional immune environment. Monoclonal antibody inhibitors may experience a pharmacokinetic advantage within the sequestered peritoneal compartment, which could allow for dosing regimens well in excess of what would be tolerated systemically. Given the exorbitant concentration of IL-6 and its soluble receptor IL-6Rα in malignant peritoneal fluid [62], we have initiated a phase I clinical trial to assess the potential of intraperitoneal infusion of the IL-6 axis antagonist tocilizumab using indwelling catheters in the peritoneal and pleural spaces of patients with malignant ascites or pleural effusions (NCT06016179). While the primary endpoints involve safety and feasibility, an extensive battery of translational companion studies is planned to assess the impact of this intervention on the cavitary and systemic immune milieu, along with any secondary signals related to palliation of ascites or anti-tumor efficacy. Intra-peritoneal blockade of the TGF-β pathway could prove to be another rational and effective strategy, given its central role in peritoneal biology, using agents currently in the developmental pipeline [52,93].

### 5.3. Intraperitoneal Vaccination

Dendritic cell and other cancer vaccination strategies have been studied in several clinical trials for GC. Guo et al., [94] reported the first case of a patient with advanced metastatic GC treated with a personalized neoantigen-loaded monocyte-derived dendritic cell (Neo-MoDC) vaccine and an immune checkpoint inhibitor. The vaccines were given alone for two months, followed by combination therapy with nivolumab. After an increase in the frequency of neoantigen-specific T cell clones in peripheral blood, tumor volume decreased, and the patient had complete regression for at least 25 months. Early attempts at *intra-peritoneal* vaccination have been sporadically reported. A phase II trial (NCT 02151448) studied the use of adjuvant dendritic cell vaccine in combination with celecoxib, IFN-α, and Rintatolimod in patients undergoing CRS and HIPEC for peritoneal metastasis [79]. Another phase II clinical trial (NTR7060) also used dendritic cell-based immunotherapy after CRS and HIPEC in patients with malignant peritoneal mesothelioma [95]. While not a vaccination strategy per se, intra-tumoral injection of adjuvants such as TLRs is also being explored. NCT 05751837 is a phase I study investigating intra-tumoral lipopolysaccharide (*Escherichia coli* 0113) in peritoneal tumors via laparoscopy. At the time of subsequent laparotomy, 14–30 days later, injected tumors are extracted and analyzed for biomarkers of immune microenvironment modulation. Although not specific to carcinomatosis of gastric origin, these early reports provide a basis for future optimization studies on regional (intra-peritoneal) delivery mechanisms and the efficacy of vaccines or adjuvants, along with combination strategies, to improve efficacy in GC-PC.

### 5.4. Oncolytic Viral Therapy

Oncolytic viral therapy in GC-PC has been pursued as a mechanism for directed intra-peritoneal immunotherapy. Currently, there are no active or completed human clinical studies using oncolytic viral therapy in GC-PC. There are, however, encouraging findings using in vitro and in vivo animal models of GC-PC. Using a murine model to study systemic delivery of reovirus into animal tumor cells, Kawaguchi et al. showed a reduction in peritoneal tumor volume and ascites [96,97]. In a separate study, RNA knockdown of phosphoglycerate kinase 1 (PGK1) through adenovirus-shPGK-1 was shown to reduce viability in human gastric adenocarcinoma cell lines [98]. Gujar et al., demonstrated that tumor-specific T cells during reovirus oncotherapy enhanced survival in tumor hosts, suggesting a synergistic response with reovirus-mediated oncolysis and the antitumor immune response in cell line models of cancer immunotherapy [99]. When combined with paclitaxel, Ishikawa et al., demonstrated that an attenuated adenovirus synergistically suppressed GC-PC [100]. Oncolytic herpes viruses selectively replicate in tumor cells and have shown promise by inducing apoptosis in gastric cancer cells [101], and decreasing peritoneal dissemination in GC-PC models [102,103]. A phase I/II clinical trial (NCT 03866525) is ongoing to determine the efficacy of a genetically engineered oncolytic herpes simplex virus type 2 in patients with malignant solid tumors [80,81]. Animal and cell culture studies to date indicate that intraperitoneal administration of oncolytic viruses may prove useful in the treatment of GC-PC [97]. 

### 5.5. Adoptive Cell Therapy

Adoptive cell therapy (ACT) is another investigational strategy for peritoneal malignancies. Bebnowska et al., have summarized theoretical targets of high priority for peritoneal CAR-T cell therapy, focusing on CEA, HER-2, EpCAM, claudin 18.2, and mesothelin [104]. Claudin 18.2-specific CAR-T cells (CT041) have been tested in a phase 1 study of 37 patients with gastrointestinal cancer, as described by Qi et al., with most patients experiencing mild cytokine release syndrome and other hematologic toxicity without dose-limiting toxicities [1]. In preliminary reports, the overall response and disease control rate for gastric cancer were 57.1% and 75%, respectively, with sustained response at 6 months in 44.8% and a six-month survival rate of >80%. EpCAM-specific CAR-T cells are also being tested for advanced peritoneal malignancies (WCH-GC-CART, NCT03563326). This phase I trial will evaluate the safety and efficacy of additional peritoneal instillation of EpCAM CAR-T cells as compared to conventional chemotherapy alone in patients with EpCAM-positive GC-PC. This study intends to recruit 40 patients and will evaluate adverse effects with secondary outcomes of overall and progression-free survival, including a pharmacokinetic evaluation of the persistence of CAR-T cells. CAR-NK cells are a distinct but related cellular therapeutic modality, with current studies using mesothelin as a CAR target. This promising line of investigation remains in pre-clinical stages at this time [82].

## 6. Conclusions and Future Directions

**Figure 1:**
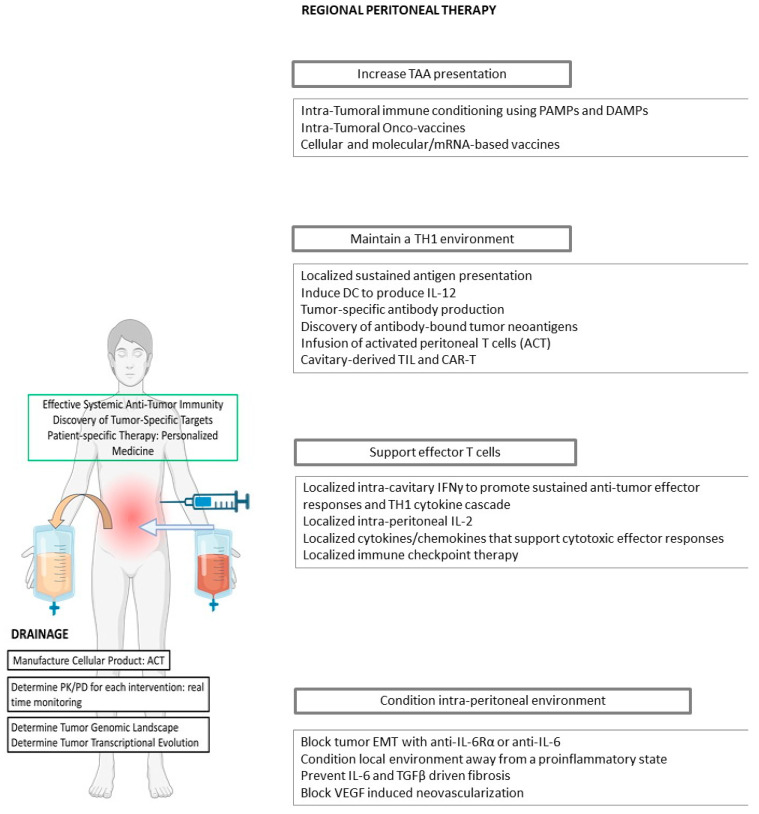
Regional immunotherapy in the peritoneal cavity. Emerging regional peritoneal options include intra-tumoral adjuvant injection, IP cytokine blockade, IP targeted therapy (monoclonal antibody or small molecule inhibitors), IP adoptive cellular therapy, and IP oncolytic viral or vaccine therapy and intra-tumoral injection. The ultimate goal of regional therapy is to generate and support a cytotoxic tumor-specific T-cell response in the peritoneal cavity. A combined or multimodal regional approach would stimulate TAA presentation by resident dendritic cells, promote an adaptive T_H_1 response while minimizing immunosuppressive or tumor-promoting mediators, and deliver ACT, oncolytics, or vaccines via regional catheters. Drainage or effluent from the peritoneal cavity could serve as a fertile source of tumor-specific ACT products, as well as biomarkers for immune status and disease response. An adaptive regional immunotherapy response could then generate systemic anti-tumor activity in extraperitoneal sites of disease, while potentially sparing patients from the systemic toxicity of immunotherapy. Abbreviations: IP—intra-peritoneal, TAA—tumor-associated antigens; PAMPs—pathogen-associated molecular pathogens; DAMPs—damage-associated molecular pathogens; TH1—Type 1 T helper cells; DC—dendritic cells; EMT—epithelial-to-mesenchymal transition; ACT—adoptive cellular therapy; TIL—tumor-infiltrating lymphocytes; PK/PD—pharmacokinetics/pharmacodynamics.

Personalized immunotherapy approaches tailored to the molecular profiles of individual patients offer the prospect of higher response rates and improved therapeutic outcomes. Systemic immunotherapy regimens, including checkpoint inhibition, have yielded disappointing results in GC-PC patients, a subset of GC with defined clinical, pathologic, molecular, and immunologic characteristics. GC-PC presents unique investigative challenges, existing as it does within the immunologically distinct, sequestered, and pro-tumorigenic peritoneal environment. Notwithstanding these limitations, we have articulated the conception of the peritoneal cavity as a bio-reactor in which modulation of the immune environment toward an adaptive phenotype through a combination of potential regional and systemic interventions could re-polarize tumor-reactive peritoneal immune cells to recognize and destroy GC-PC (Figure 1). The peritoneal cavity and malignant ascites are rich sources of tumor-reactive lymphocytes, and targeted optimization strategies for this regional milieu are intended to create favorable conditions for the re-infusion of ex vivo-activated native lymphocytes or the infusion of adoptive cell therapy products (CAR-T or CAR-NK cells). Under these circumstances, the peritoneal fluid could also serve as a source of biomarkers of response and, it is hoped, serve as an epicenter from which durable systemic responses could be achieved as tumor-reactive lymphocytes migrate back into the systemic circulation from the peritoneal cavity. Well-designed, stepwise pre-clinical and early-phase clinical trials will likely be necessary to tackle this complex problem, but recent advances in knowledge of GC-PC biology and peritoneal immuno-biology provide hope that effective regimens are within reach to improve oncologic outcome and quality of life in GC-PC patients.

## Figures and Tables

**Table 1:** Summary of Ongoing Clinical Trials Regarding Immunotherapy for Gastric Cancer.

Clinical Trial	Researchers	Phase	Treatment Protocol	Patients (N)	Outcome Measures	Preliminary Results
*Systemic Checkpoint Inhibition*						
NCT 05648487 (HIPEC-10)	Lei et al.	Phase 2	HIPEC combined with Sintilimab	46	Rate of R0 resection, OS, ORR, EFS, RFS	Not yet recruiting
NCT 05204173 (DRAGON-09)	Yuan et al. [77]	Phase 2	Sintilimab, IP and IV PTX, plus oral S-1	36	One-year survival rate, R0 resection rate, three-year OS, three-year PFS	Recruiting
*Intraperitoneal Immunotherapy*						
NCT 04222114	Qi et al. [78]	Phase 3	IP Catumaxomab vs. localized supportive treatment	282	OS, PFS, progression free interval of peritoneal metastatic lesions	Recruiting
NCT 06016179	Wagner, et al.	Phase 1	Four incremental weekly intra-cavitary doses of tocilizumab	12	Successful intra-cavitary administration of tocilizumab; adverse events	Recruiting
*Intraperitoneal Vaccination*						
NCT 02151448	Ramanathan et al. [79]	Phase 1, 2	Adjuvant alpha-DC1 vaccine combined with celecoxib, IFNα, and rintatolimod in CRS/HIPEC patients	64	Recommended phase 2 dose, adverse events, TTP, OS, PFS	Alpha-DC1 vaccine is not appropriate for patients undergoing CRS/HIPEC
NCT 05751837	Wagner et al.	Phase 1	Injection of LPS into one abdominal tumor	6	Safety and tolerability	Recruiting
*Oncolytic viral therapy*						
NCT 03866525	Zhang et al. [80,81]	Phase 1, 2	OH2 injection with or without irinotecan or HX008	300	DLT, MTD, biodistribution and biologic effect of OH2, anti-tumor activity and immunogenicity of OH2	Intratumoral injection of OH2 was well-tolerated with a no DLTs and MTD
*Adoptive Cell therapy*						
NCT 03563326 (WCH-GC-CART)	Cao et al. [82]	Phase 1	EpCAM CAR-T cells vs. chemo	40	Adverse effects of EpCAM CAR-T, OS, metabolism kinetics of CAR-T cells, PFS	Recruitment status unknown

Abbreviations: N—number, HIPEC—heated intraperitoneal chemotherapy, OS—overall survival, ORR—objective response rate, EFS—event-free survival, RFS—relapse-free survival, IP—intraperitoneal, IV—intravenous, PTX—paclitaxel, PFS—progression-free survival, DC—dendritic cell, IFN—interferon, CRS—cytoreductive surgery, TTP—time to progression, LPS—lipopolysaccharide, OH2—oncolytic type 2 herpes simplex virus, HX008—anti-PD1 antibody, DLT—dose-limiting toxicity, MTD—maximum-tolerated dose.
